# Supplementary material for: Distinct regulation of ATM signaling by DNA single-strand breaks and APE1
Source: Nat Commun. 2024 Aug 7;15:6517. doi: 10.1038/s41467-024-50836-6 (PMC11306256; doi:10.1038/s41467-024-50836-6)
Supplement: Supplementary file 1 — Supplementary Information [file 41467_2024_50836_MOESM1_ESM.pdf]

Supplementary Information

Distinct regulation of ATM signaling by DNA single-strand breaks and APE1

Haichao Zhao[1], Jia Li[1], Zhongsheng You[2], Howard D. Lindsay[3], Shan Yan[1,4,5*]

[1]Department of Biological Sciences, University of North Carolina at Charlotte; Charlotte, NC 28223, USA
[2]Department of Cell Biology and Physiology, Washington University School of Medicine; St. Louis, MO 63110, USA
[3]Lancaster Medical School, Faculty of Health and Medicine, Lancaster University; Lancaster LA 4YQ, UK
[4] School of Data Science, University of North Carolina at Charlotte; Charlotte, NC 28223, USA
[5]Center for Biomedical Engineering and Science, University of North Carolina at Charlotte; Charlotte, NC 28223, USA

[*]Corresponding author. Email: shan.yan@charlotte.edu

The SI file includes:

Fig. S1
Fig. S2
Fig. S3
Fig. S4
Fig. S5
Table S1

Supplementary Figures and Legends

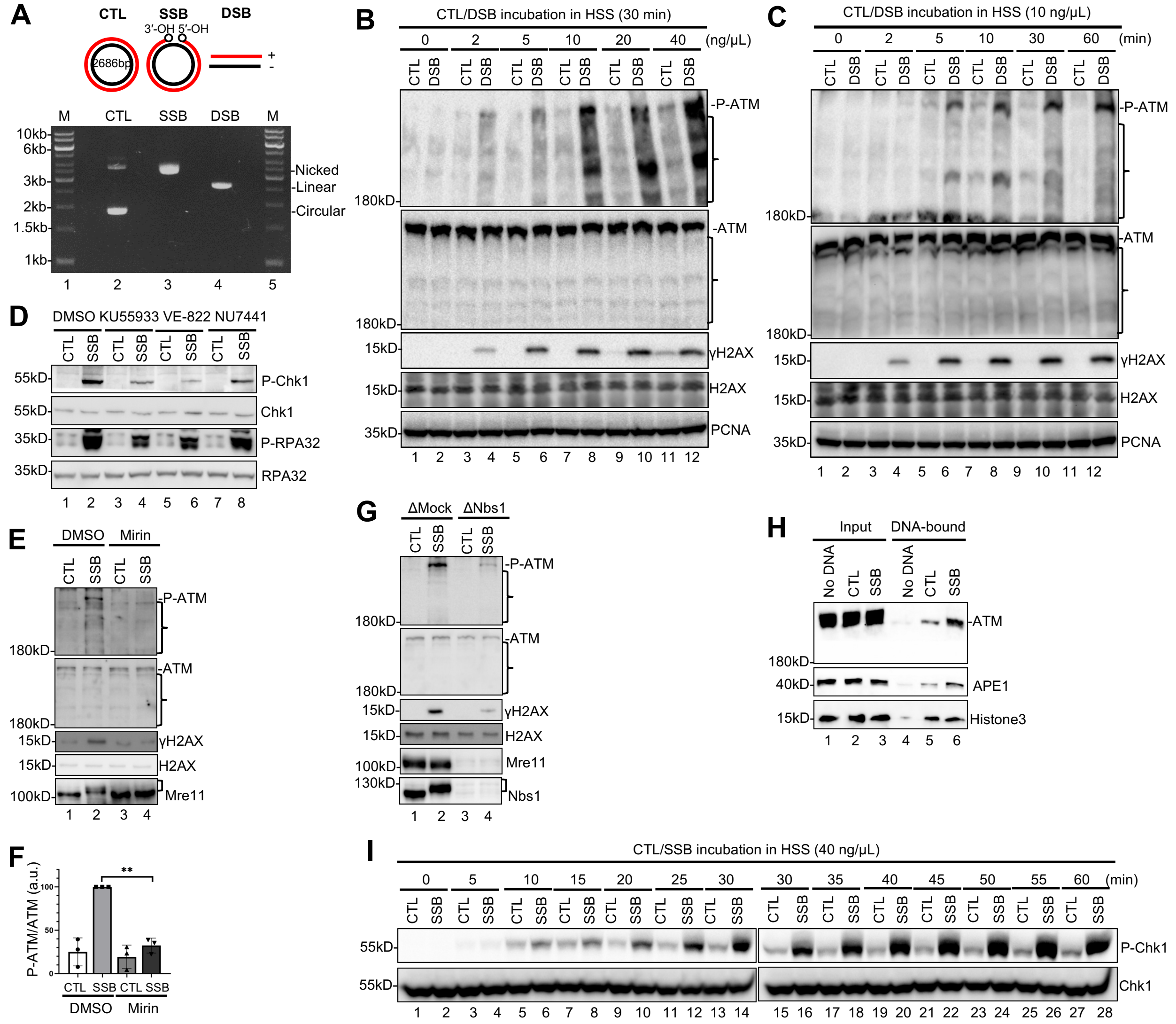

Fig. S1. Characterization of the ATM DDR activation by DSB and SSB plasmids in the HSS (Related to Fig. 1).

(**A**) Quality control of CTL, SSB and DSB plasmid. (B) CTL or DSB plasmid was added to HSS at different concentrations as indicated, and incubated for 30 min. Extracts were examined via immunoblotting analysis as indicated.

(**C**) CTL or DSB plasmid was added to HSS at a final concentration of 10 ng/μL. After different times of incubation at room temperature, the extracts were examined via immunoblotting analysis.

(**D**) ATM inhibitor KU55933 (1mM), ATR inhibitor VE-822 (1mM), or DNA-PK inhibitor NU7441 (25μM) was added to HSS supplemented with CTL or SSB plasmid (40 ng/μL). After a 15-min incubation, total egg extracts were examined via immunoblotting analysis as indicated.

(**E**) Mre11 inhibitor Mirin (100μM) was added to HSS supplemented with CTL or SSB plasmid (40ng/μL). After incubation for 15 min, the extracts were examined via immunoblotting.

(**F**) Quantification and statistical analysis of P-ATM vs total ATM from experiments shown in Panel (E). a.u., arbitrary units. Data are presented as mean values ± SD. ** p=0.0054 (two-tailed, paired t-test). n=3.

(**G**) CTL or SSB plasmid was added to Mock- or Nbs1-depleted HSS. After a 15-min incubation, the egg extracts were examined via immunoblotting analysis as indicated.

(**H**) H_2O (No DNA), CTL or SSB plasmid (40ng/μL) was added to HSS. After room temperature incubation for 10 min, the total egg extracts ("Input") and DNA-bound fractions were examined via immunoblotting.

(**I**) CTL or SSB plasmid was added to HSS at a final concentration of 40ng/μL. After different times of incubation at room temperature, the extracts were examined via immunoblotting analysis for Chk1 phosphorylation (P-Chk1) and Chk1.

The data presented in Panel **B**-**D** and **G**-**I** are representative of three biological replicates.] in Panel **E** and **G** indicates mobility shift of Mre11 or Nbs1. } indicates non-specific bands. Source data are provided as a Source Data file.

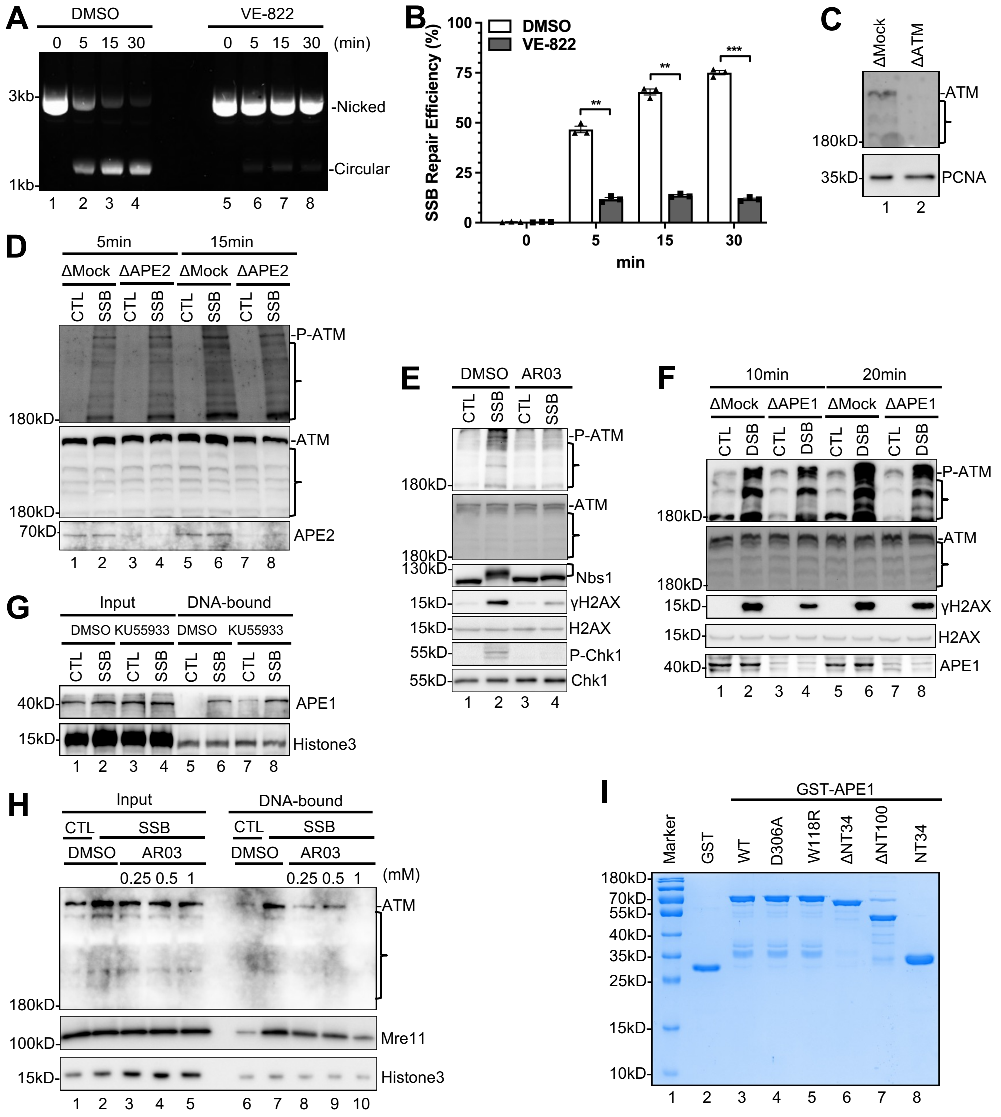

Fig. S2. Characterization of SSB repair and regulatory mechanisms of SSB-induced ATM activation in the HSS system (Related to Fig. 2).

(**A**) SSB plasmid was added to HSS supplemented with DMSO or ATR inhibitor VE-822 (1mM). After different incubation times, the DNA repair products were isolated and analyzed on an agarose gel.

(**B**) Quantification and statistical analysis of experiment results shown in (A). Data are presented as mean values ± SD. ***p*(5min) =0.0030; ***p*(15min) =0.0016; ****p*(30min)=0.0003; two-tailed, paired t-test, n=3.

(**C**) The efficiency of ATM depletion from HSS was examined via immunoblotting.

(**D**) CTL or SSB plasmid was added to Mock- or APE2-depleted HSS. After different incubation times (5 and 15 min), the egg extracts were examined via immunoblotting analysis as indicated.

(**E**) CTL or SSB plasmid was added to HSS supplemented with AR03 at a final concentration of 1mM. After incubation for 15 min at room temperature, the egg extracts were examined via immunoblotting analysis as indicated.] indicates mobility shift of Nbs1.

(**F**) CTL or SSB plasmid was added to HSS supplemented with AR03 at different concentrations (0.25mM, 0.5mM and 1mM). After a 5-min incubation, the total egg extracts and DNA-bound fractions were examined via immunoblotting.

(**G**) CTL or SSB plasmid was added to HSS supplemented with KU55933 (1mM). After 10 min incubation, the total egg extracts and DNA-bound fractions were examined via immunoblotting. Histone3 was used as loading control.

(**H**) CTL or DSB plasmid was added to Mock- or APE1-depleted HSS. After different incubation times (10 and 20 min), the egg extract was examined via immunoblotting analysis as indicated.

(**I**) Coomassie blue staining of mutants and truncations of GST-APE1 recombinant proteins.

The data presented in Panel **A**, and **C**-**I** are representative of three biological replicates. } indicates non-specific bands. Source data are provided as a Source Data file.

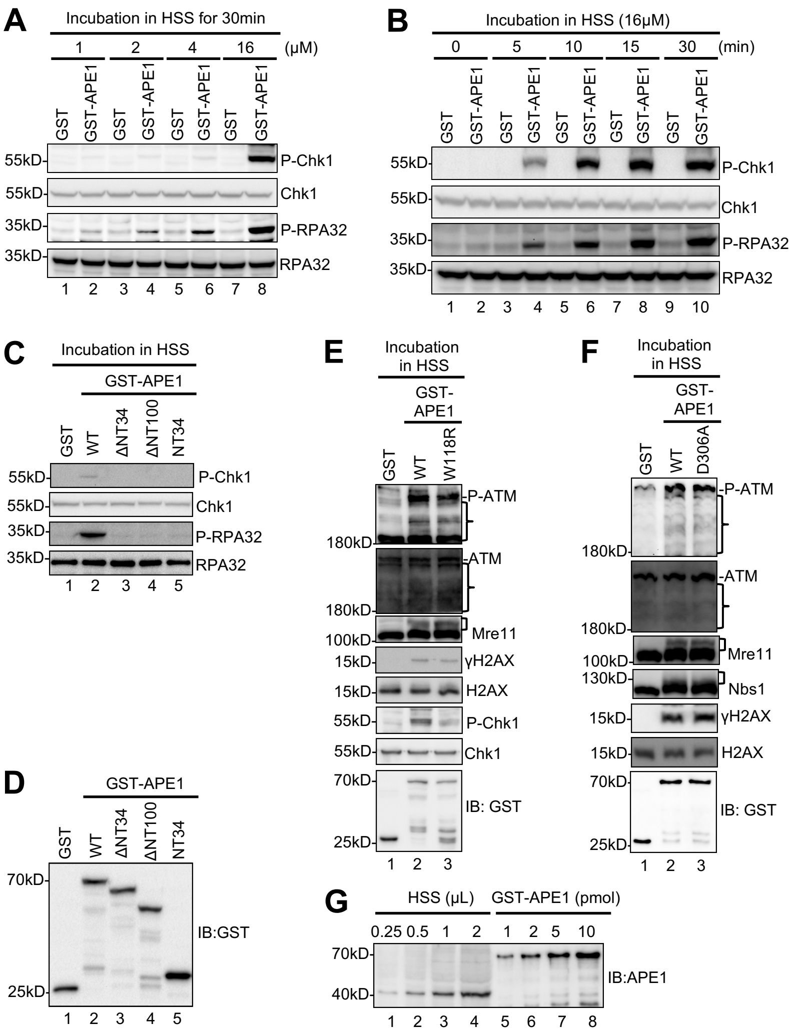

Fig. S3. Purified GST-APE1 protein also triggered ATR DDR in the HSS system (Related to Fig. 3).

(**A**) GST or GST-APE1 was added to HSS at different concentrations as indicated and incubated for 30 min. Extracts were examined via immunoblotting analysis for ATR pathway as indicated.

(**B**) GST or GST-APE1 was added to HSS at a final concentration of 16μM. After different time of incubation at room temperature, the extracts were examined via immunoblotting analysis for ATR pathway as indicated.

(**C**) GST, GST-APE1-WT, GST-APE1-ΔNT34 (AA35-316), GST-APE1-ΔNT100 (AA101-316) or GST-APE1-NT34 (AA1-AA34) was added to HSS at a final concentration of 16μM and incubated for 30 min. Extracts were examined via immunoblotting analysis for ATR signaling as indicated.

(**D**) Related to Fig. 3E and S3C. Extracts were also examined via immunoblotting analysis for GST to ensure the addition of GST-fused proteins were equal.

(**E**) GST, GST-APE1-WT, or GST-APE1-W118R was added to HSS at a final concentration of 16μM and incubated for 30 min. Extracts were examined via immunoblotting analysis for ATM and ATR DDR pathways as indicated.

(**F**) GST, GST-APE1-WT, or GST-APE1-D306A was added to HSS at a final concentration of 16μM and incubated for 30 min. Extracts were examined via immunoblotting analysis for ATM DDR pathway as indicated.

(**G**) Different volumes of HSS and different quantities of recombinant GST-APE1 proteins were examined via immunoblotting analysis using anti-APE1 antibodies.

The data presented are representative of three biological replicates.] in Panel **E** and **F** indicates mobility shift of Mre11 or Nbs1. } indicates non-specific bands. Source data are provided as a Source Data file.

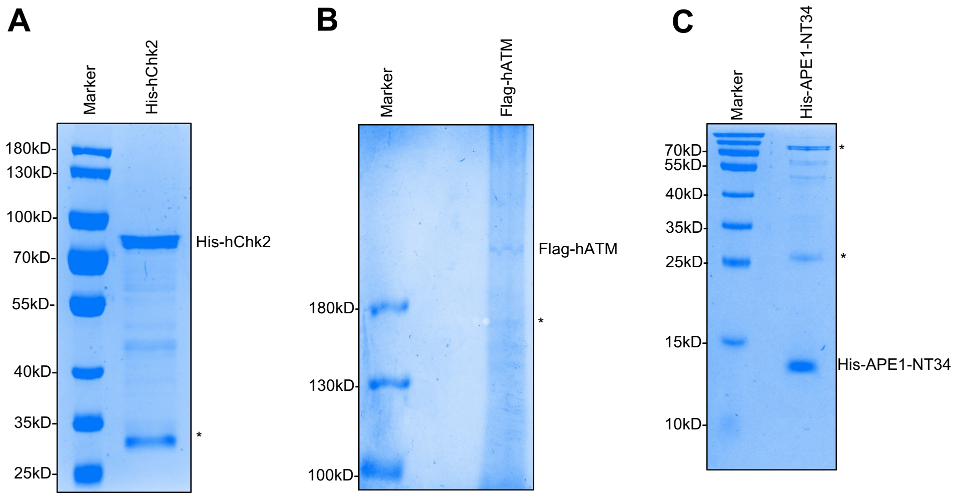

Fig. S4. SDS PAGE analysis of purified recombinant proteins (Related to Fig. 4).
(**A**) Coomassie blue staining of His-hChk2 recombinant protein.
(**B**) Coomassie blue staining of Flag-hATM recombinant protein.
(**C**) Coomassie blue staining of His-APE1-N34 recombinant protein.
*indicates some potential contaminating or degradation products in the preparation. The data presented are representative of three biological replicates. Source data are provided as a Source Data file.

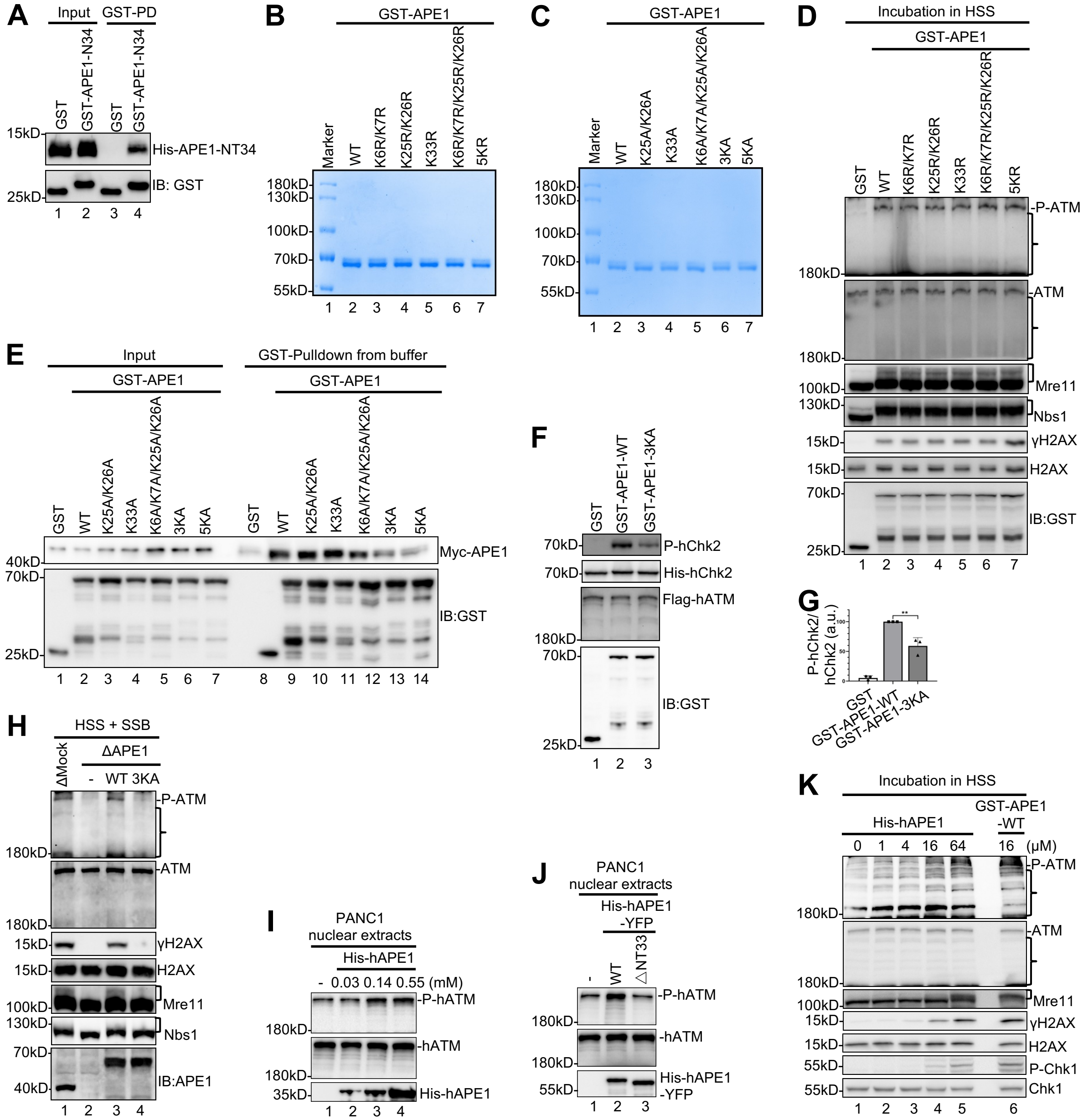

Fig. S5. Characterization of various WT/mutant GST-APE1 protein in ATM DDR activation and kinase assays (Related to Fig. 5).

(**A**) GST and ΔNT34 GST-APE1 were examined for interaction with His-APE1-NT34 in the interaction buffer. Input and GST-PD (pulldown) samples were examined via immunoblotting analysis using anti-His and anti-GST antibodies.

(**B**) Coomassie blue staining of GST-APE1 K-R mutant recombinant proteins.

(**C**) Coomassie blue staining of GST-APE1 K-A mutant recombinant proteins.

(**D**) GST, WT, K6R/K7R, K25R/K26R, K33R, K6R/K7R/K25R/K26R, 5KR was added to HSS at a final concentration of 16μM, and incubated for 30 min. Extracts were examined via immunoblotting analysis for ATM DDR pathway as indicated.

(**E**) GST, WT, K25A/K26A, K33A, K6A/K7A/K25A/K26A, 3KA, or 5KA GST-APE1was examined for interaction with WT Myc-APE1 in the interaction buffer. Input and bead-bound fractions were examined via immunoblotting analysis for Myc and GST.

(**F**) Kinase assays were performed with GST, GST-APE1-WT or 3KA, Flag-hATM and His-hChk2.

Phosphorylation was visualized with anti-Chk2-P–T68.

(**G**) Quantification and statistical analysis of the ratio of P-hChk2 vs. hChk2 from (F). Data are presented as mean values ± SD. ***p*=0.0071 (two-tailed, unpaired t-test). n=3.

(**H**) SSB plasmid was added to Mock-depleted or APE1-depleted HSS supplemented with negative control, WT and 3KA Myc-APE1 protein. After a 20-min incubation, the total egg extracts were examined via immunoblotting.

(**I**) Increased concentrations of purified His-hAPE1 was added to PANC1 nuclear extracts for a 30-min incubation. The total samples were examined via immunoblotting analysis.

(**J**) Purified WT or ΔNT34 His-hAPE1 protein (0.55mM) was added to PANC1 nuclear extracts for a 30-min incubation. The total samples were examined via immunoblotting analysis.

(**K**) His-hAPE1 protein or GST-APE1 protein was added to HSS at different concentrations as indicated, and incubated for 30 min. Extracts were examined via immunoblotting analysis for ATM pathway as indicated.

The data presented in Panel **A**-**F** and **H**-**K** are representative of three biological replicates.] in Panel **D**, **H** and **K** indicates mobility shift of Mre11 or Nbs1. } indicates non-specific bands. Source data are provided as a Source Data file.

Table S1. DNA sequences of oligos used in this work.

Primer ID	Sequence	Source	Identifier
pGEX-4T1-xAPE1 (F)	5'-GGGGGGGATCCATGCCCAAGAGAGGGAAGAAG-3'	[63]	N/A
pGEX-4T1-xAPE1 (R)	5'-GGGGGCTCGAGTTATATGGCCATCAAGAGTG-3'	[63]	N/A
pGEX-4T1-xAPE1-ΔNT34 (F)	5'-GGGGGGGATCCGCAAAAGAGCCAGAACCAGTTG-3'	[63]	N/A
pGEX-4T1-xAPE1-ΔNT100 (F)	5'-GGGGGGGATCCAAATTGCTGCCTCCAGATG-3'	[63]	N/A
pGEX-4T1-xAPE1-NT34 (R)	5'-GGGGGCTCGAGTTAAGCCTTCCCTGCTCCCTTCTT-3'	This paper	N/A
pGEX-4T1-xAPE1-W118R (F)	5'GAATACCCCCACAAATACCGGGCATGCCCTGATGAAAAG-3'	This paper	N/A
pGEX-4T1-xAPE1-W118R (R)	5'CTTTTCATCAGGGCATGCCCGGTATTTGTGGGGGTATTC-3'	This paper	N/A
pCS2+MT-xAPE1 (F)	5'-GGGGGCCATGGAGATGCCCAAGAGAGGGAAGAAG-3'	[63]	N/A
pCS2+MT-xAPE1 (R)	5'-GGGGGCTCGAGTTATATGGCCATCAAGAGTG-3'	[63]	N/A
pCS2+MT-xAPE1-ΔNT34 (F)	5'GGGGGCCATGGAGGCAAAAGAGCCAGAACCAGTTG-3'	This paper	N/A
pCS2+MT-xAPE1-ΔNT34 (R)	5'-GGGGGCTCGAGTTATATGGCCATCAAGAGTG-3'	This paper	N/A
pET-28a-xAPE1-NT34 (F)	5'-GGGGGGGATCCATGCCCAAGAGAGGGAAGAAG-3'	This paper	N/A
pET-28a-xAPE1-NT34 (R)	5'-GGGGGCTCGAGTTAAGCCTTCCCTGCTCCCTTCTT-3'	This paper	N/A
pET-28a-hChk2-D347A (F)	5'-GGGAAGCTTGCATGTCTCGGG AGTCGGATGTTG-3'	This paper	N/A
pET-28a-hChk2-D347A (R)	5'-GGGCTCGAGTCAACACAGCAGCACACACAG-3'	This paper	N/A
pGEX-4T1-xAPE1-K6R/K7R (F)	5'CCATGCCCAAGAGAGGGAGGAGGGAAGAAGTTTGTGCTGC-3'	This paper	N/A
pGEX-4T1-xAPE1-K6R/K7R (R)	5'GCAGCACAAACTTCTTCCCTCCTCCCTCTCTTGGGCATGG-3'	This paper	N/A
pGEX-4T1-xAPE1-K25R/K26R (F)	5'GGAATGAACCGGAAGTTAGGAGGGGGAAGAAGGGAGCAGG-3'	This paper	N/A
pGEX-4T1-xAPE1-K25R/K26R (R)	5'-CCTGCTCCCTTCTTCCCCCTCCTAACTTCCGGTTCATTCC-3'	This paper	N/A
pGEX-4T1-xAPE1-K33R (F)	5'GAAGAAGGGAGCAGGGAGGGCTGCAAAAGAGCCAG-3'	This paper	N/A
pGEX-4T1-xAPE1-K33R (R)	5'-CTGGCTCTTTTGCAGCCCTCCCTGCTCCCTTCTTC-3'	This paper	N/A
pGEX-4T1-GST-xAPE1-K6R/K7R/K25R/K26R/K33R (F)	5'GGGGGAAGAAGGGAGCAGGGAGGGCTGCAAAAGAGCCAGAACC-3'	This paper	N/A
pGEX-4T1-GST-xAPE1-K6R/K7R/K25R/K26R/K33R (R)	5'GGTTCTGGCTCTTTTGCAGCCCTCCCTGCTCCCTTCTTCCCCC-3'	This paper	N/A
pGEX-4T1-xAPE1-K6A/K7A (F)	5'CCATGCCCAAGAGAGGGGCGGCGGAAGAAGTTTGTGCTGC-3'	This paper	N/A

pGEX-4T1-xAPE1-K6A/K7A (R)	5’GCAGCACAAACTTCTTCCGCCGCCCCTCTCTTGGGCATGG-3’	This paper	N/A
pGEX-4T1-xAPE1-K25A/K26A (F)	5’GGAATGAACCGGAAGTTGCGGCGGGGAAGAAGGGAGCAGG-3’	This paper	N/A
pGEX-4T1-xAPE1-K25A/K26A (R)	5’CCTGCTCCCTTCTTCCCCGCCGCAACTTCCGGTTCATTCC-3’	This paper	N/A
pGEX-4T1-xAPE1-K33A (F)	5’GGGGAAGAAGGGAGCAGGGGCGGCTGCAAAAGAGCCAGAACCAG-3’	This paper	N/A
pGEX-4T1-xAPE1-K33A (R)	5’CTGGTTCTGGCTCTTTTGCAGCCGCCCCTGCTCCCTTCTTCCCC-3’	This paper	N/A
