# Supplementary material for: Distinct regulation of ATM signaling by DNA single-strand breaks and APE1
Source: Nat Commun. 2024 Aug 7;15:6517. doi: 10.1038/s41467-024-50836-6 (PMC11306256; doi:10.1038/s41467-024-50836-6)
Supplement: Supplementary file 3 — Reporting Summary [file 41467_2024_50836_MOESM3_ESM.pdf]

nature portfolio

Corresponding author(s): Shan Yan

Last updated by author(s): Jul 15, 2024

Reporting Summary

Nature Portfolio wishes to improve the reproducibility of the work that we publish. This form provides structure for consistency and transparency in reporting. For further information on Nature Portfolio policies, see our Editorial Policies and the Editorial Policy Checklist.

Statistics

For all statistical analyses, confirm that the following items are present in the figure legend, table legend, main text, or Methods section.

n/a	Confirmed	
☐	☒	The exact sample size (n) for each experimental group/condition, given as a discrete number and unit of measurement
☐	☒	A statement on whether measurements were taken from distinct samples or whether the same sample was measured repeatedly
☐	☒	The statistical test(s) used AND whether they are one- or two-sided<br>*Only common tests should be described solely by name; describe more complex techniques in the Methods section.*
☒	☐	A description of all covariates tested
☒	☐	A description of any assumptions or corrections, such as tests of normality and adjustment for multiple comparisons
☐	☒	A full description of the statistical parameters including central tendency (e.g. means) or other basic estimates (e.g. regression coefficient) AND variation (e.g. standard deviation) or associated estimates of uncertainty (e.g. confidence intervals)
☐	☒	For null hypothesis testing, the test statistic (e.g. F, t, r) with confidence intervals, effect sizes, degrees of freedom and P value noted<br>*Give P values as exact values whenever suitable.*
☒	☐	For Bayesian analysis, information on the choice of priors and Markov chain Monte Carlo settings
☒	☐	For hierarchical and complex designs, identification of the appropriate level for tests and full reporting of outcomes
☒	☐	Estimates of effect sizes (e.g. Cohen's d, Pearson's r), indicating how they were calculated

Our web collection on statistics for biologists contains articles on many of the points above.

Software and code

Policy information about availability of computer code

Data collection	Image Lab software (Bio-Rad, version 6.0.1) with standard settings was used to measure IB blot intensities.
Data analysis	GraphPad Prism (version 9) was used for statistical analysis. Photoshop (version 25.9.1) and Microsoft Office (Powerpoint version 16.78.3; Word version 16.78.3) were used for data processing and analysis.

For manuscripts utilizing custom algorithms or software that are central to the research but not yet described in published literature, software must be made available to editors and reviewers. We strongly encourage code deposition in a community repository (e.g. GitHub). See the Nature Portfolio guidelines for submitting code & software for further information.

Data

Policy information about availability of data

All manuscripts must include a data availability statement. This statement should provide the following information, where applicable:

- Accession codes, unique identifiers, or web links for publicly available datasets
- A description of any restrictions on data availability
- For clinical datasets or third party data, please ensure that the statement adheres to our policy

All data are available in the main text or the Supplementary Information. The original Western blot data and quantifications of blot bands and associated statistical analysis data are provided in the Source Data file with this paper. Requests for the materials generated in this study should be directed to the corresponding author (SY).

Research involving human participants, their data, or biological material

Policy information about studies with human participants or human data. See also policy information about sex, gender (identity/presentation), and sexual orientation and race, ethnicity and racism.

Reporting on sex and gender	This study does not involve human participants so no such information is collected.
Reporting on race, ethnicity, or other socially relevant groupings	This study does not involve human participants so no such information is collected.
Population characteristics	This study does not involve human participants so no such information is collected.
Recruitment	This study does not involve human participants so no such information is collected.
Ethics oversight	This study does not involve human participants so no such information is collected.

Note that full information on the approval of the study protocol must also be provided in the manuscript.

Field-specific reporting

Please select the one below that is the best fit for your research. If you are not sure, read the appropriate sections before making your selection.

[x] Life sciences [ ] Behavioural & social sciences [ ] Ecological, evolutionary & environmental sciences

For a reference copy of the document with all sections, see nature.com/documents/nr-reporting-summary-flat.pdf

Life sciences study design

All studies must disclose on these points even when the disclosure is negative.

Sample size	No sample size calculation was performed. Sample size predetermination is not applicable to this study, which doesn't involve analyses of individual cells.
Data exclusions	No data were excluded from the analyses.
Replication	The data presented are representative of three biological replicates unless otherwise specified. All attempts at replication were successful.
Randomization	Samples were randomly allocated to different groups.
Blinding	Blinding was not possible or not relevant to this study. In addition to appropriate internal positive and negative controls, samples were treated (e.g., doses and times) following proper experimental designs. Our practice was based on our published research and that of others.

Reporting for specific materials, systems and methods

We require information from authors about some types of materials, experimental systems and methods used in many studies. Here, indicate whether each material, system or method listed is relevant to your study. If you are not sure if a list item applies to your research, read the appropriate section before selecting a response.

Materials & experimental systems

n/a	Involved in the study	
[ ]	[x]	Antibodies
[ ]	[x]	Eukaryotic cell lines
[x]	[ ]	Palaeontology and archaeology
[ ]	[x]	Animals and other organisms
[x]	[ ]	Clinical data
[x]	[ ]	Dual use research of concern
[x]	[ ]	Plants

Methods

n/a	Involved in the study	
[x]	[ ]	ChIP-seq
[x]	[ ]	Flow cytometry
[x]	[ ]	MRI-based neuroimaging

Antibodies

Antibodies used	All the antibodies information has been described in the section of "Methods" and also here: Anti-Xenopus APE1 and APE2 custom antibodies were described previously61,63. Custom antibodies against ATM, Mre11, Nbs1, and RPA32 and were described previously19,21,118. Antibodies against ATM phosphorylation at Ser1981 (Rockland Immunochemicals, Cat#200-301-500), Chk1 (Santa Cruz Biotechnology, Cat#sc-8408), Chk1 phosphorylation at Ser345 (Cell Signaling Technology, Cat#2348), Chk2 (Santa Cruz Biotechnology, Cat#sc-9064), Chk2 phosphorylation at Thr68 (Santa Cruz Biotechnology, Cat#sc-16297-

	R), Flag (Thermo Fisher, Cat#MA1-91878), GST (Santa Cruz Biotechnology, Cat#sc-7898), H2AX (Cell Signaling Technology, Cat#7631S), H2AX phosphorylation at Ser139 (Cell Signaling Technology, Cat#2577S), H3 (Abcam, Cat#ab1791), His (Santa Cruz Biotechnology, Cat#sc-8036), Myc (Santa Cruz Biotechnology, Cat#sc-40), RPA32 phosphorylation at Ser33 (Bethyl Laboratories, Cat#A300-246A), and PCNA (Santa Cruz Biotechnology, Cat#sc-56) were purchased from commercially available vendors.
Validation	More validation information was described in the section "Immunoblotting analysis and antibodies" with appropriate references in the section of "Methods".

Eukaryotic cell lines

Policy information about cell lines and Sex and Gender in Research

Cell line source(s)	HEK293 (ATCC #CRL-1573), WT/APE1-KO HEK293FT (Kind gift from Dr. Bruce Demple and Dr. Dmitry Zharkov), and PANC1 (ATCC #CRL-1469) cell lines.
Authentication	Cell authentication was confirmed by STR profiling method.
Mycoplasma contamination	All cells were negative for mycoplasma contaminations.
Commonly misidentified lines (See ICLAC register)	No commonly misidentified cell lines were used in the study.

Animals and other research organisms

Policy information about studies involving animals; ARRIVE guidelines recommended for reporting animal research, and Sex and Gender in Research

Laboratory animals	Female African clawed frogs (Xenopus laevis) with age of ~4-12 years were purchased from NASCO.
Wild animals	Wild animals are not applicable.
Reporting on sex	Eggs derived from female frogs were used to make the HSS. Male frogs could not be used for preparing Xenopus egg extracts.
Field-collected samples	This is not applicable.
Ethics oversight	The care and use of Xenopus laevis followed established protocols approved by the Institutional Animal Care and Use Committee at the University of North Carolina at Charlotte (IACUC Protocol-22-023).

Note that full information on the approval of the study protocol must also be provided in the manuscript.

Plants

Seed stocks	This is not applicable to this study.
Novel plant genotypes	This is not applicable to this study.
Authentication	This is not applicable to this study.
